# Evaluation of gold fiducial marker manual localisation for magnetic resonance-only prostate radiotherapy

**DOI:** 10.1186/s13014-018-1029-7

**Published:** 2018-06-05

**Authors:** Matteo Maspero, Peter R. Seevinck, Nicole J. W. Willems, Gonda G. Sikkes, Geja J. de Kogel, Hans C. J. de Boer, Jochem R. N. van der Voort van Zyp, Cornelis A. T. van den Berg

**Affiliations:** Universitair Medisch Centrum Utrecht, Heidelberglaan 100, Utrecht, 3508 GA The Netherlands

**Keywords:** Magnetic resonance imaging, Radiotherapy treatment planning, MR-only treatment planning, Pre-treatment positioning, Fiducial marker localization, Manual detection, Accuracy, Precision

## Abstract

**Background:**

The use of intraprostatic gold fiducial markers (FMs) ensures highly accurate and precise image-guided radiation therapy for patients diagnosed with prostate cancer thanks to the ease of localising FMs on photon-based imaging, like Computed Tomography (CT) images. Recently, Magnetic Resonance (MR)-only radiotherapy has been proposed to simplify the workflow and reduce possible systematic uncertainties. A critical, determining factor in the accuracy of such an MR-only simulation will be accurate FM localisation using solely MR images.

**Purpose:**

The aim of this study is to evaluate the performances of manual MR-based FM localisation within a clinical environment.

**Methods:**

We designed a study in which 5 clinically involved radiation therapy technicians (RTTs) independently localised the gold FMs implanted in 16 prostate cancer patients in two scenarios: employing a single MR sequence or a combination of sequences. Inter-observer precision and accuracy were assessed for the two scenarios for localisation in terms of 95% limit of agreement on single FMs (LoA)/ centre of mass (LoA _CM_) and inter-marker distances (IDs), respectively.

**Results:**

The number of precisely located FMs (LoA <2 mm) increased from 38/48 to 45/48 FMs when localisation was performed using multiple sequences instead of single one. When performing localisation on multiple sequences, imprecise localisation of the FMs (3/48 FMs) occurred for 1/3 implanted FMs in three different patients. In terms of precision, we obtained LoA _CM_ within 0.25 mm in all directions over the precisely located FMs. In terms of accuracy, IDs difference of manual MR-based localisation versus CT-based localisation was on average (±1 STD) 0.6 ±0.6 mm.

**Conclusions:**

For both the investigated scenarios, the results indicate that when FM classification was correct, the precision and accuracy are high and comparable to CT-based FM localisation. We found that use of multiple sequences led to better localisation performances compared with the use of single sequence. However, we observed that, due to the presence of calcification and motion, the risk of mislocated patient positioning is still too high to allow the sole use of manual FM localisation. Finally, strategies to possibly overcome the current challenges were proposed.

**Electronic supplementary material:**

The online version of this article (10.1186/s13014-018-1029-7) contains supplementary material, which is available to authorized users.

## Background

The use of intraprostatic fiducial markers (FMs) ensures highly accurate and precise image-guided radiation therapy (IGRT) for patients diagnosed with prostate cancer [1]. Specifically, it has been shown that markers enable for a safe reduction of PTV margin [2–5]. To accurately position patients based on the target location, a set of three non-co-linear markers centred in the prostate is the minimum requirement allowing triangulation and measurement of the prostate position in different planes [6]. Most markers are made of inert metals (gold and titanium, for example). For prostate cancer, gold FMs are generally employed and their use allows accurate prostate localisation [7] on photon-based images thanks to their high density, which increases radio opacity [8]. Markers have usually a cylindrical shape, a diameter ranging between 0.5 and 1.5 mm and length between 2 and 10 mm; they remain in the patient permanently [7]. To ensure geometrically accurate IGRT treatments, FMs are localised on computed tomography (CT) during treatment simulation and they are localised on kV/MV imaging before patient irradiation to verify and eventually match the pre-treatment patient position with the planned position [9, 10]. The localisation is performed based on a distinct image contrast the FMs induce in the CT as well as kV/MV images [11]. The distinctness of the contrast ensures that false classification of FMs, e.g. due the presence of calcifications, is unlikely.

Recently, magnetic resonance imaging (MRI) and its superior soft tissue contrast with respect to photon-based imaging [12, 13] enabled more accurate delineation of the prostate [14–16]. To exploit the advantages offered by this imaging modality, the use of MRI in radiotherapy planning is rapidly expanding [6]. Nowadays, the treatment simulation is generally based on CT and MRI images. Before treatment target delineation, CT and MR images are rigidly registered based on the location of FMs on both image modalities [17, 18]. The accuracy of the registration is generally considered as being within 1 mm [19].

More recently, MRI-based radiotherapy - also called “MR-only” radiotherapy - has been proposed [20–22] to reduce systematic spatial uncertainties introduced when registering CT and MRI images [23]. Moreover, an MR-only workflow would reduce costs of the treatment and patient exposure to ionising radiation [24]. Additionally, MR-only treatment planning is particularly desirable in the context of MR-guided photon [25–27] and eventually proton [28–30] radiotherapy. On the other hand, the introduction of an MR-only radiotherapy pathway raises a series of challenges as enabling MR-based dose calculations [22], dealing with distortions in MR images [31], and the use of MR images as reference for position verification purposes [32].

Up to now, research on such an MR-only workflow has mainly focused upon generation of so-called synthetic-CT images to allow dose calculation based on MR image information alone. Less attention has been paid to the issue of MR-based FM localisation, which is a major determining factor in obtaining accurate radiation treatment of prostate cancer.

In MRI, FMs are depicted as signal voids in magnitude images since they do not produce nuclear magnetic resonance signal [33]. The appearance of FM voids varies according to imaging parameters [34] and the FM orientation with respect to the magnetic field [35]. Up to now, to minimise manual interaction, automated MR-based FM localisation methods have been proposed [36–39]. These methods are promising, resulting in acceptable accuracy and relatively high detection rates ranging from 84% to 96%. However, we can not rely on the fact that all FMs will be automatically localised, leaving to manual observers the burden to correct for missed detections. These missed detections can derive from misclassifying blood clots or calcification as FMs since they all appear as signal voids in MR images [7, 40]. Therefore, in addition to the requirement of achieving a high localisation accuracy, the risk of misclassification of signal voids (i.e. false positives) should be very low as this could result in systematic errors in patient positioning [41].

In current clinical practice, MR-based manual FM localisation is performed by radiation therapy technicians (RTTs) for registering MR to CT images [17, 41]. In such a setting, the presence of CT images greatly aids the RTT to discern whether signal voids in MR images can be classified as FM, calcifications or blood clots.

While this MR-based FM localisation for CT/MR simulation is clinically accepted, no previous study has investigated the reliability of a solely MR-based FM localisation by manual observers, which is the expected scenario within MR-only radiotherapy.

The aim of this study is to evaluate the performance of manual FM localisation during the planning of prostate cancer patients’ external beam radiotherapy treatments. Furthermore, since in a clinical environment multiple sequences are usually available, we aim at investigating whether the use of multiple sequences may impact the manual FM localisation. We conducted a study to assess the inter-observer precision and accuracy of the MR-based FM localisation among RTTs. Furthermore, we evaluated in our patients’ group the occurrence of misclassification of FMs.

## Methods

This study is divided into three parts. First, we selected patients and acquired CT and MR images (Patient preparation and selection section). Second, we performed a multi-observer manual MR-based localisation (MR-based fiducial marker localisation section). Finally, we evaluated the precision and accuracy of the manual FM localisation and investigated whether the observer agreement may lead to a precise and accurate patient alignment (Statistical analysis section).

### Patient preparation and selection

The study was performed on patients with prostate carcinoma diagnosis who underwent radiotherapy planning at the University Medical Center Utrecht (The Netherlands) between September and October 2015. The study has been conducted in accordance with regulations from the local ethical committee.

For position verification purposes, each patient received three intraprostatic cylindrical gold FMs (HA2 Medizintechnik GmbH, Germany) measuring 1 mm (diameter) by 5 mm (length). The FMs were transperineally implanted under ultrasound guidance by a physician prior to the imaging session using two 18-gauge needles placed with the aid of a template.

Patient positioning at CT scan (Brilliance CT Big Bore, Philips Medical Systems, Cleveland, Ohio, USA) was conducted simulating the treatment, i.e. using a flat table, knee wedges, positioning arms on the chest and tattooing the patient with the aid of laser alignment.

Patient setup at 3T MR scan (Ingenia Omega HP, Philips Healthcare, Best, The Netherlands) was performed using a knee wedge, but without a flat table top, without positioning arms on the chest and without laser-aided positioning. Patients were scanned using anterior and posterior phased array coils (dS Torso and Posterior coils, 28 channels, Philips Healthcare, Best, The Netherlands). To avoid compression of patients’ anatomy, two in-house-built coil bridges supported the anterior coil. The location of FMs from CT images was obtained as previously described in [39]. No rectum or bladder preparation protocol was applied before imaging sessions, but the patients were asked to empty their rectum in case rectal filling was noticed being larger than 5 cm during the imaging session.

Criteria for selecting the subjects were: patients had gold FMs implanted prior to the imaging sessions, patients underwent CT and MRI on the same day acquiring three specific MR sequences (see next section for further details) and were without metallic implants. CT scans were performed with the following imaging parameters: 120 kV, exposure time = 923 ms, tube current between 121 and 183 mA, in-plane matrix = 512x512 pixels, and 3 mm slice thickness. The resolution was variable depending on the field of view (FOV) used. The typical size of the FOV was 500x500x300 mm^3^, which corresponds to an in-plane resolution of 0.98x0.98 mm^2^.

### MR-based fiducial marker localisation

Among the acquired MR images, we tested manual FM localisation on the three following sequences, for which imaging parameters are reported in Table 1:

1. a 3D Cartesian balanced steady-state free precession (bSSFP) sequence with spectral attenuated inversion recovery to obtain fat suppression and highlight prostate boundaries. The images acquired with this sequence were used by the physician to perform prostate delineations. The vendor’s name for this sequence was “3D balanced turbo field echo”.

**Table 1:** Imaging parameters of the sequences used for gold FM manual localisation: the second column provides the details for the balanced steady-state free precession (bSSFP) sequence, the third column for the radio frequency spoiled gradient-recalled echo (SPGR) sequence acquired right after the bSSFP and the fourth column for the gradient-recalled echo (GRE) sequence acquired at the end of the examination

Imaging parameters	bSSFP	SPGR	GRE
TE_1_/(TE_2_)/TR [ms]	1.98/3.96	1.4/2.7/4.4	1.4/2.7/4.6
Flip Angle [°]	40	10	10
FOV^a^ [mm^3^]	250x250x90	467x467x300	449x449x90
Acquisition Matrix^a^	252x234x90	312x314x200	376x376x75
Reconstruction Matrix^a^	512x512x90	320x320x200	400x400x75
Reconstructed Voxel^a^ [mm^3^]	0.5x0.5x1.0	1.5x1.5x1.5	1.1x1.1x1.2
Bandwidth [Hz/voxel]	945	1078	1142
Readout direction	AP	AP	AP
Acquisition time	4 min 29 s	4 min 1 s	2 min 10 s

^a^expressed in terms of anterior-posterior, right-left and superior-inferior directions

The term FOV refers to field of view, AP to anterior-posterior
2. a 3D Cartesian T1-weighted dual radio frequency spoiled gradient-recalled echo (SPGR) sequence. The SPGR sequence was acquired right after the bSSFP and used by the physician to distinguish bleedings from the primary lesion. The vendor’s name for this sequence was “3D T1 fast field echo”.
3. a 3D Cartesian dual gradient-recalled echo (GRE) sequence. This sequence was acquired at the end of the examination to have an independent sequence for FM localisation. The field of view (FOV) was reduced to the sole target reducing acquisition time as well as making this sequence less prone to motion artefacts. The vendor’s name for this sequence was “3D fast field echo”.

The sequences were selected as best candidates for FM identification after inspection of previously acquired patient images. The rationale underlying the choice was the high contrast between FM location and surrounding tissues and the spatial high resolution (voxel size <1.2x1.2x1.5 mm^3^). Furthermore, they were expected to preserve geometrical accuracy thanks to the 3D acquisition and large bandwidth (>900 Hz/pixel); similar sequences were used also by other institutions for FM localisation [37, 42]. Five RTTs independently performed manual localisation of FMs by identifying the top and bottom of the markers on magnitude images of the bSSFP sequence. The RTTs were requested to follow standardised instructions regarding zoom and window/level of the images. The provided instructions are available as part of the Additional file 1. The RTTs involved in the study had varying experience as technicians: 11, 6.5, 5, 5 and 10 years. They all had experience with position verification. Four RTTs (all the observers except the first observer), had experience with image registration between MR and CT images of: 0, 2, 2, 2.5 and 2.5 years, respectively. They were new to MR-only FM localisation since this procedure was initiated with this study. The observers were asked to report for which patients the FM localisation was perceived as being problematic and they got the freedom to provide three or four candidates. Also, the RTTs indicated which FM was the most difficult to distinguish. The FM centre was calculated as the mean position between the manually identified top and bottom positions. Note that the observed length of the FM may not correspond to the nominal length of the FM (5mm). The term “apparent” location was used to refer to the location of top and bottom as identified by the observers. Note that apparent location of the top and bottom of each FM coincided with the centre of a voxel, thus, the calculated centre of the FMs may be located with resolution higher than a single voxel.

To investigate whether the use of multiple sequences impacts the FM localisation, the RTTs performed the FM localisation again using multiple MR images after localisation using images of the sole bSSFP sequence. In particular, in this second localisation the RTTs employed images of the bSSFP, the second echo of the SPGR and the GRE sequences. Note that the location of the markers on the bSSFP was not made available when repeating the localisation. In the case of inter-scan FM motion, the RTTs were instructed to consider the position of the FMs on the bSSFP as a reference.

For completeness, the following metrics were also recorded: the apparent length as characterised in each sequence and by the different observers, the time required by each observer to complete the FM localisation and the number of FMs for which the localisation was perceived as being problematic. These results are reported as part of the Additional file 2.

### Statistical analysis

The analysis was performed on the FM centres as located using both a “single” sequence (bSSFP) and “multiple” MRI sequences (bSSFP, SPGR and GRE). In case four FMs were identified, the FM located with the lowest reliability was excluded from the statistical analysis. To generate a consistent FM marker labelling among observers, FMs were numbered (from 1 to 3) according to the position of their centre along the superior-inferior direction. In case FMs were located in the same transverse plane, the left-right direction was used for labelling. To keep the consistency of the labelling among the observers, the labelling was manually checked and, when necessary, corrected. The analysis was performed in Matlab (R2015a, the MathWorks Inc., Natick, Massachusetts, United States).

#### Inter-observer agreement or spatial precision

##### Single FM locations

For each observer, the number of detected FMs was reported. An agreement position was defined as the mean position among all the five observers. To assess the precision among the five observers, FM locations were compared calculating a 95% limit of agreement (= 1.96 times the standard deviation (STD) [43]) of the distance to the mean position (LoA) in the three directions (X = anterior-posterior, Y = right-left and Z = superior-inferior). For comparison with [44], a threshold for clinical acceptability was set to 95% LoA ≤2 mm. Bar plots providing a visual assessment of the inter-observer variability were created reporting also a more stringent LoA threshold of 1 mm. An investigation of the CT and MR images was performed on an individual patient basis to investigate the causes underlying imprecise localisation of FMs; schematic representations of the inter-observer localisation were also examined.

##### Centre of mass locations

To verify the impact on patient alignment, the location of the centre of mass (CM) among all the FMs was calculated for each patient and observer. For each patient, the 95% limit of agreement (= 1.96 times the standard deviation (STD) [43]) with the average position of the CMs (LoA _CM_) was calculated among all the observers in the three directions [44]. To assess agreement of the CM position among the observers a threshold of LoA _CM_<2 mm was used for comparison with [44]. To verify clinical CM agreement, the threshold LoA _CM_<1 mm was employed. Bar plots providing a visual assessment of the inter-observer agreement variability were created. Note that patient alignment is generally performed on the centre of mass location [41]; therefore, this is considered as the final metric to assess inter-observer precision.

#### Intra-observer agreement

To evaluate whether, over all the observers, a statistically significant variation of FM location occurred between localisation using only the bSSFP sequence and the combination of bSSFP, SPGR and GRE sequences, we performed Wilcoxon rank-sum test at the confidence level of 95% in the three directions on LoA and LoA _CM_.

#### Spatial accuracy

The difference of inter-marker distances (IDs) between the FMs located in CT and MRI were calculated for the precisely located (LoA <2 mm) markers using single and multiple sequences. For each observer and over all the observers, the absolute difference between the ID of MRI and CT were calculated as in [38] and characterised in terms of mean, median, standard deviation (STD) and range ([minimum, maximum]).

## Results

Seventeen consecutive prostate patients (61.4-81.9 years, mean age = 68.7 years, median age = 68.3 years, inter quartile range (IQR) = 66.1-70.8 years) were considered for inclusion in the study. All the patients were staged as T1c-3b, Gleason score ≥ 6 and one of the patients (P14) had a hip implant and was excluded from the analysis (the localisation and images for this patient are presented in the Additional file 3 in Fig. 3)). Within the patient population, the average prostate volume during imaging sessions was 56.8 ml (range = 32.1-117.3 ml, median volume = 54.8 ml, IQR = 42.9-70.9 ml) and body mass index was on average 26.4 kg/m^2^ (range = 19.9-30.7 kg/m^2^, IQR = 24.9-28.7 kg/m^2^). No patient received adjuvant hormonal therapy.

Patients underwent intensity-modulated radiotherapy, using 5 beams of 10 MV, with a prescribed dose of 77 Gy to the entire prostate in 35 fractions (2.2 Gy per fraction). Other clinical prescriptions are specified in [45]. Each of the patients had three FMs implanted leading to a total of 48 FMs and 580 FM localisations (16 patients x 3 FMs x 5 observers x 2 sequence scenarios) performed over all the observers. The FMs were implanted at least one week prior to imaging.

**Fig. 1:**
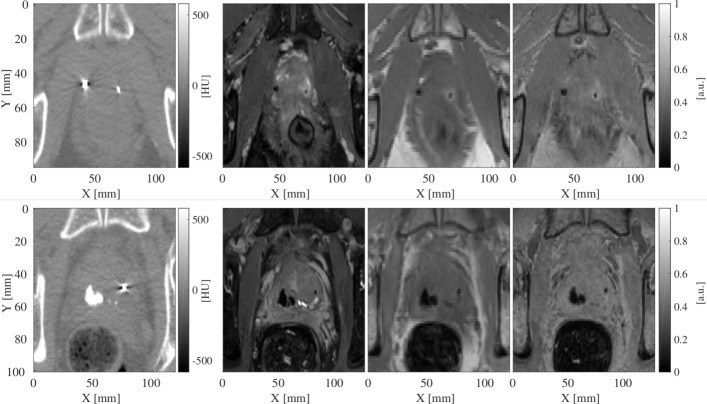
Zoom of an axial slice of CT (left), bSSFP (centre-left), SPGR (centre-right) and GRE (right) images for patients P1 (top) and P9 (bottom) before image registration. The axes X and Y indicate the anterior-posterior and right-left directions. The intensity of CT image is in Hounsfield Units (HU), while of MR images is normalised to the maximum over the whole dataset. Note the presence of calcifications for the patient P9; they are visible as high intensity on CT and signal void on MR images

During the pre-planning imaging session, MRI scans were performed within maximum 70 min (mean time = 45 min, minimum time of 20 min and IQR = 34-50 min) after the CT scans. For all the patients, the bSSFP and SPGR sequences were acquired one after each other with a maximum time difference of 5 min, while the GRE sequence was acquired at least 15 min after the SPGR sequence. Figure 1 shows a zoomed axial slice of CT, bSSFP, SPGR and GRE images for a patient in which all observers agreed on the locations of the FMs (P1, top) and a patient in which one FM was challenging (P9, bottom)

### Single FM locations

**Fig. 2:**
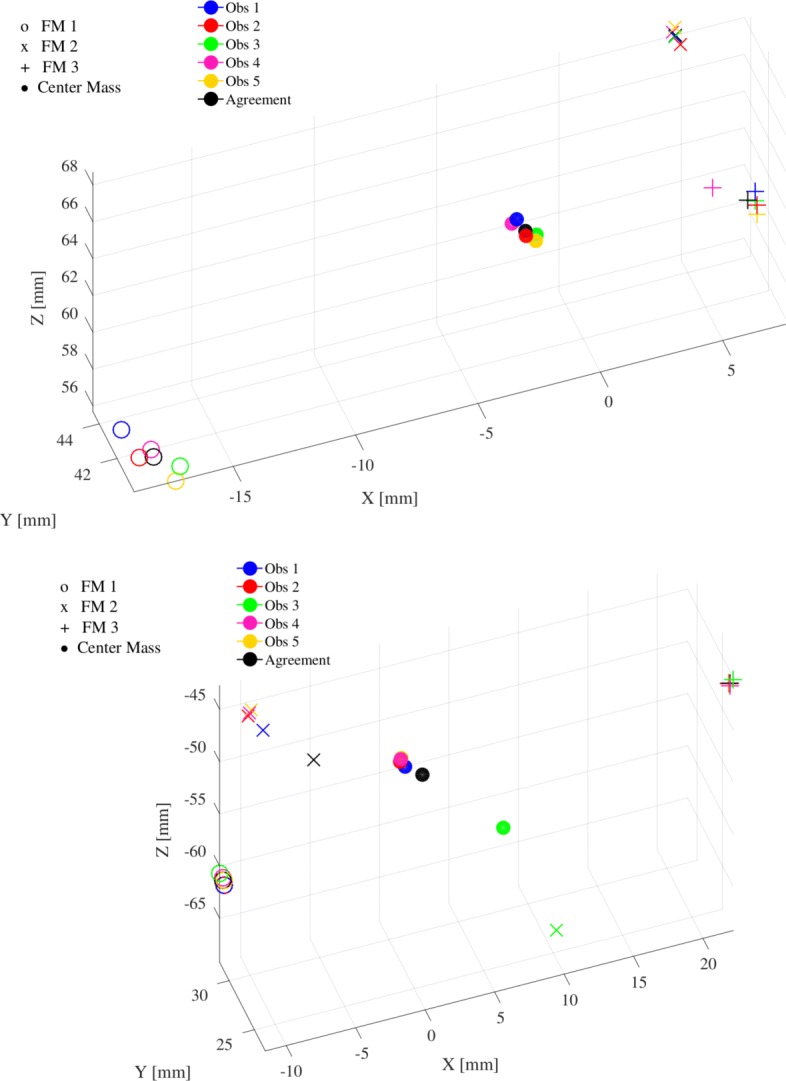
Schematic representation of the centres of the FMs as localised by all the observers for patient P1 (top) and P9 (bottom) using multiple sequences (bSSFP, SPGR and GRE). The labelling of the FM is indicated by the marker: ∘, x, + and ∙ for FM having number 1, 2, 3 and for agreement position, respectively. For patient P1 (top), the LoA _CM_ of FM1 over the five RTTs was 0.27, 0.31 and 0.38 mm in X, Y and Z, respectively, which was considered clinically acceptable; for patient P9 (bottom), the LoA _CM_ for FM2 over the five observers was 11.17, 0.99 and 13.70 mm in X, Y and Z, respectively, which was considered clinically unacceptable

All the observers detected three FMs for all the patients, except one observer (Obs1) who detected four FMs for two patients (P4 and P17) when using the bSSFP sequence. When also the SPGR and GRE sequences were employed, all the RTTs localised three FMs. Figure 2 provides a schematic representation of the centres of the FMs as localised by all the observers for patients P1 and P9 using multiple sequences. The agreement position is as well shown. As example, taking into consideration the FM with the largest spread for patient P1 (FM 1), the LoA were 0.69, 0.57, 0.84 mm in X (anterior-posterior), Y (right-left) and Z (superior-inferior) directions, respectively.

**Fig. 3:**
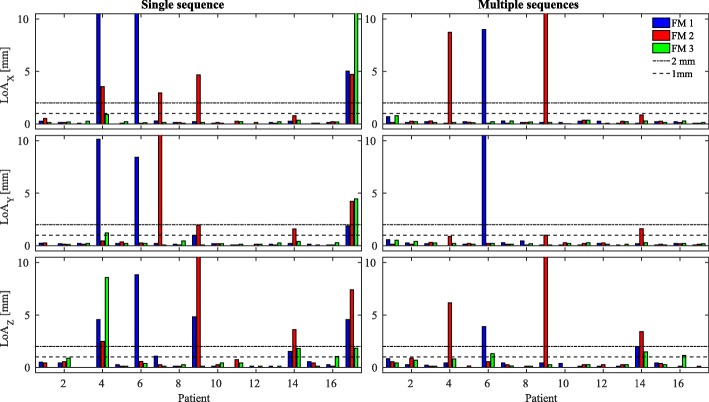
The 95% limit of agreement (LoA) calculated, for each patient, over the five observers for the single fiducial marker (FM) in the three directions, where X = anterior-posterior (top), Y = right-left (center) and Z = superior-inferior (bottom). On the left is shown the FM localisation as performed using the bSSFP sequence only, while on the right the FM localisation as performed using multiple sequences. The dotted and dashed lines represent the LoA of 2 mm, while the dotted lines represent the LoA of 1 mm. Note that patient P14 had hip implant and the results are here presented but were excluded in the statistical analysis

The bar plots of LoA for all the patients over the five RTTs is shown in Fig. 3. The LoA was found to be higher than 2 mm in one of the three directions for 10/48 and 3/48 FMs when the observers located on a single (bSSFP) and multiple (bSSFP, SPGR and GRE) sequences, respectively. This resulted in an increased agreement (45/48) when the observers located on multiple sequences with respect to a single sequence (38/48). Over all the three directions, the Wilcoxon rank-sum test at 95% confidence interval resulted in significantly different LoA when comparing FM location obtained with one or multiple sequences. In particular, as shown in Fig. 3 with the use of multiple sequences for two patients (P7 and P17) the LoA decreased below 2 mm. When localising using a single sequence over all the patients, LoA was found to be >2 mm for more than one FM per patient, while when localisation was performed on multiple sequences LoA was >2 mm only for one FM per patient. Focusing on the scenario with the largest agreement (localisation performed using multiple sequences), localisation of maximum one FM was found to be imprecise for 3/16 patients: P4, P6 and P9. Excluding these 3 FMs (considering, therefore, 45/48 FMs), the average (±1 STD) LoA was 0.19 ±0.15, 0.18 ±0.12 and 0.30 ±0.31 mm in anterior-posterior, right-left and superior-inferior directions, respectively. After the investigation of the images acquired for the patients resulting in an imprecise FM localisation, we observed that patients P4 (Fig. 1 in Additional file 3) and P9 (Figs. 1 and 2 bottom) were characterised by the presence of large (>2 mm in diameter) intra-prostatic calcifications. In both cases, 1/5 RTTs (Obs1 for P4 and Obs3 for P9) localised one of the FMs far away from the other four observers. Figure 2 shows that a misclassification occurred for patient P9 when considering the FM2 and Obs3. The same occurred for FM2 and Obs1 for patient P4 (Fig. 1 in Additional file 3 bottom). After observing the location of the misclassified FMs as reported by the two observers in the MR and CT images, we found that the FMs were located in correspondence of calcifications. For one patient (P6, as shown in Additional file 3 in Fig. 2), one of the FMs was not visible on bSFFP but appeared on SPGR and GRE; we hypothesised that motion reduced the visibility of the FM on the bSSFP impacting reliability of the localisation for this FM. Considering the results from a different perspective, for the total 240 (16 patients x 5 observers x 3 FMs) single observer localisations using multiple sequences, 2 times calcifications were marked as FMs by one of the RTTs and no agreement could be found for one FM among all the five RTTs. This would result in misclassification for 7 out of the 240 single observer localisations, or 7/80 (16 patients x 5 observers) single observer localisations of the CM in the case the outliers cannot be eliminated.

### Center of mass locations

**Fig. 4:**
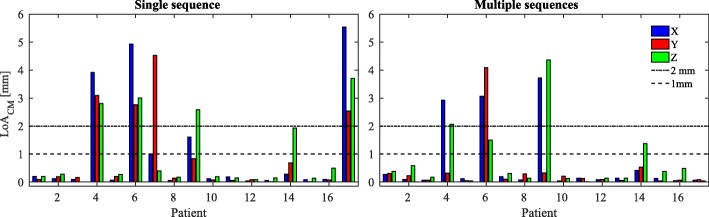
The 95% limit of agreement of the centre of mass (LoA _CM_) calculated, for each patient, over the five observers for a single fiducial marker (FM) in the three directions, where X = anterior-posterior (blue), Y = right-left (red) and Z = superior-inferior (green). On the left is shown the localisation of the CM as performed using the bSSFP sequence only, while on the right the localisation of the CM as performed using multiple sequences. The dotted and dashed lines represent the LoA of 2 mm, while the dotted lines represent the LoA of 1 mm. Note that patient P14 had hip implant and the results are here presented but were excluded in the statistical analysis

Figure 4 presents the bar plot of the 95% LoA _CM_ for all the patients over the five RTTs. The LoA _CM_ was found to be >1 mm in one of the three directions (X, Y or Z) for 5/16 and 3/16 patients when the observers located on a single (bSSFP) and multiple (bSSFP, SPGR and GRE) sequences, respectively. Over all the three directions, the Wilcoxon rank-sum test at 95% confidence interval resulted in significantly different LoA when comparing FM locations obtained with one or multiple sequences. Excluding the imprecisely located CMs, the average (±1 STD) LoA _CM_ when localisation was performed with single sequence was 0.10 ±0.05, 0.10 ±0.06 and 0.19 ±0.13 mm in anterior-posterior, right-left and superior-inferior directions, respectively; the average (±1 STD) LoA _CM_ when localisation was performed with multiple sequences was 0.11 ±0.06, 0.13 ±0.09 and 0.23 ±0.18 mm in anterior-posterior, right-left and superior-inferior directions, respectively. In all the directions, the average LoA _CM_ is <0.25 mm.

### Spatial accuracy

Table 2 shows the mean, median, STD and range of the absolute difference in the ID of the precisely located FMs (LoA <2 mm) using single and multiple sequences. Among all the observers, the average ID difference is slightly lower (0.5 ±0.6 mm) when locating with multiple sequences with respect to with a single sequence (0.7 ±0.6 mm).

**Table 2:** The mean, median, standard deviation (STD) and range ([min, max]) of the absolute difference in the inter-marker distances (IDs) of the precisely located FMs between CT and MRI for all the single observers and for all the five observers

Sequence	Observer	Mean	Median	STD	Range
Single	1	0.8	0.6	0.7	[0.1, 3.1]
	2	0.6	0.5	0.5	[0.0, 2.5]
	3	0.7	0.6	0.5	[0.0, 2.1]
	4	0.7	0.6	0.6	[0.1, 2.9]
	5	0.7	0.5	0.6	[0.1, 2.5]
	All	0.7	0.6	0.6	[0.0, 3.1]
Multiple	1	0.7	0.4	0.6	[0.0, 2.7]
	2	0.6	0.4	0.6	[0.0, 3.0]
	3	0.7	0.5	0.7	[0.0, 2.5]
	4	0.7	0.5	0.6	[0.0, 2.8]
	5	0.7	0.6	0.5	[0.0, 2.5]
	All	0.6	0.5	0.6	[0.0, 3.0]

The results were calculated excluding 10/48 and 3/48 FMs for the localisation performed on a single and multiple sequences, respectively. All the values are expressed in mm

## Discussion

The precision and accuracy of manual localisation of intraprostatic gold FMs using solely MR images was evaluated in the context of an MRI-only simulation workflow. Note that this fundamentally differs from localisation of FMs on MR images in the current CT-MR simulation workflow for registration purposes as the CT images can be used to minimise misclassification on the MR images. In this sense, this study was conducted to verify whether an MR-only simulation could facilitate a robust positioning workflow comparable to current CT-based positioning in all the cases.

In this study, the use of multiple sequences led to precise localisation (LoA <2 mm) in more patients and of more FMs (13/16 patients and 45/48 FMs) than localisation with a single sequence only (11/16 patients and 38/48 FMs). For both scenarios, the precision calculated as the average of LoA _CM_ in all the directions on the precise localised FMs was within 0.25 mm. The results are in good agreement with others [18, 44, 46, 47]. Huisman et al. [18] obtained a precision of 0.5 mm in the centroid of the prostate on a cohort of 21 patients when assessing registration of CT and MR images. Ullman et al. [46] reported a mean inter-observer variability of 0.9 ±0.6 mm when performing registration on photon-based portal images. Deegan et al. [47] reported that inter-observer LoA on the applied registration, which is comparable to the LoA _CM_, was in the range of about ±2 mm. Literature reporting single FM localisation precision has not been found.

In general, in our study, when FMs were precisely localised, they were also accurately localised. In particular, we found an inter-observer accuracy of 0.7 mm with the single sequence and of 0.6 mm with the multiple sequences. These results are slightly more accurate than what presented when comparing a human observatory to automatic FM localisation by Gustafsson et al. [38] and in line with the accuracy previously considered acceptable for the CM localisation performed with photon-based imaging (0.6 mm) [48].

However, for a single FM in 3/16 cases, precise localisation was not achieved. That implies that a correct positioning in these patients can not be guaranteed. Based on the thorough investigation of the images of these specific patients, we concluded that the following two causes may have led to imprecise FM localisation: (1) presence of calcifications miscalssified as FM and (2) motion during the bSSFP sequence. (1) Previous studies reported the presence of calcifications in 40 to 88% of prostate cancer patients [7, 38, 40, 49]. In our study, for 2/16 patients the presence of calcifications led to misclassified FM localisation for 1/5 RTT. Interestingly, the observers seemed to be aware of the difficulties and they reported that the localisation procedure for such patients was problematic (see Fig. 1 in the Additional file 2). (2) Motion as a possible cause of hampered accuracy of FM localisation has already been reported in the literature for the bSSFP sequence [42]. The readout of this sequence was 3D leading to typical acquisition times of 2–3 min, and thus motion blurring is likely to occur.

To obtain accurate localisation for all the patient cases, we believe that redundancy should be added in the localisation procedure to lower the risk of FM misclassification. In this sense, we foresee the following as possible ways to increase the redundancy:

- *multiple observers localisation*. Whenever an RTT would have low confidence in the FM localisation, an independent observer could perform localisation and assess a posteriori the initially found position. In this scenario, the experience of the RTT may influence the outcome. Further investigations are necessary to evaluate whether such scenario will lead to accurate localisation in all the cases.
- *implantation of a fourth marker*. The use of a fourth FM could be easily performed without increasing the patient discomfort: the fourth FM could be collinearly placed with the third FM avoiding a new needle insertion. In case of FM misclasssification, the RTTs may explicitly exclude one of the FM when correcting patient set-up, remaining with a sufficient number of FMs to enable the procedure. On the other hand, with four FMs several permutations of 3 FMs could be considered and the RTTs would need consistently choose the FMs between imaging modalities to obtain identical set-up corrections.
- *resorting to automatic localisation*. Given the promising result obtained with automatic gold FM localisation methods [36–39], resorting to a combination of manual and automated MR-based FM localisation methods may ensure safe MR-based simulation of patient position.

In our institution further investigation is ongoing to verify that using automatic localisation [39] is a viable approach including also the insertion of a fourth FM. Alternatively to the redundancy options above proposed, another centre [42] reported that using kV radiography after FM implantation provided independent images that facilitated MR-based FM localisation. Similarly to this approach, we could also speculate about designing a workflow that foresees referring the patients to CT in case of dubious manual FM localisation at the MR scan. Performing a low dose CT for all the patients for the sole purpose of FM localisation could be another possibility.

Strategies to possibly solve FM misclassification other than adding redundancy may involve 1) further MR sequence optimisation and 2) employing different MR sequences. 1) Further MR sequence optimisation could, for example, be employed to diminish the susceptibility to motion by reducing the acquisition time of the employed sequences. In addition, sequence optimisation may impact visualisation and also manual localisation performance. 2) Among the available MRI sequences, only the images of one echo of the gradient-echo sequences have been taken into consideration in this study. It may occur that acquiring with different image parameters or MR sequences may result in more favourable manual FM localisation performance. For example, recently, the use of multi-echo images showed promising results, thanks to the increasing size of a signal void when increasing the echo time [38]; Future studies could investigate whether MR sequence optimisation or the use of other sequences may be more suitable for FM localisation, verifying accuracy and precision performance.

From a general perspective, in our study, five RTTs were involved, making the findings representative of a realistic situation. As the observers were not familiar with MR-only FM localisation, it may be expected that better results may be obtained by training the observers for this specific context. In this sense, it may be interesting to verify, in a future study, the influence of clinical experience on manual localisation performance.

Comparing our study to previous research, a limitation of the presented cohort is its size, although, no other research has been presented to assess manual FM localisation with such details and reporting localisation performances within a realistic clinical environment. Recently, Gustafsson et al. [38] presented results of the accuracy of manual FM localisation and a larger cohort (44 patients). Unfortunately, the precision has not been reported.

Recently, the use of MR-visible fiducial markers have been proposed offering new possibilities for MR-based marker localisation [34, 50]. In addition, FM localisation may also be based on mechanisms other than imaging. For example, it has been shown that transponders can be safely implanted ensuring real-time prostate localisation [51]. Both these approaches may be adopted in an MR-only workflow offering an alternative to gold FM localisation.

In the perspective of MR-only Radiotherapy, and considering the case of gold FM, the use of multiple sequences would enable manual marker localisation for precise and accurate simulation of prostate cancer patients’ position prior to irradiation in almost all the cases. Nevertheless, believing that an MR-only simulation should facilitate a robust positioning workflow, we think the risk of mislocated patient positioning is still too high and that additional redundancy is essential to enable a safe clinical practice.

## Conclusion

We studied inter-observer precision and accuracy of manual gold FM localisation for MR-only prostate cancer external beam therapy simulation over five RTTs for two scenarios: employing a single MRI sequence (bSSFP) or a combination of multiple sequences (bSSFP, SPGR and GRE). The use of multiple sequences (bSSFP, SPGR and GRE) led to better localisation performances compared with the use of a single sequence (bSSFP). For both the scenarios, the results indicate that when FM classification was correct, the precision and accuracy are high and comparable to CT-based FM localisation. However, the risk of mislocated patient positioning due to FM misclassification is still too high to allow the sole use of manual FM localisation. For future work, we hypothesise that further increasing redundancy by increasing the number of FM per patient and by setting up a system to rely on multiple observations or automatic localisation is necessary to increase the detection rate and enable clinical introduction.

## Additional files

Additional file 1 [1.] *GeneralGuidelineFMloc.pdf* which presents a short description of the procedure;
[2.] *PracticalInstructionFMloc.pdf* which describes step-by-step the procedure;
[3.] *Checklist_Obs.pdf* which is aimed at supporting the RTTs during the procedure in keeping track and annotate for which patient the localisation was found problematic. (ZIP 194 kb)

Additional file 2 Annotations on the FM localisation. As part of the supplementary material, we report the apparent length of the FMs for each observer and the time spent by each observer performing the FM localisation over all the patients. In particular, Table 1 shows the mean, standard deviation (STD), range [min, max] of the apparent length, expressed in mm. The weighted mean over all the observer is 7.5±0.6 mm and 7.7±0.7 mm for localisation using a single and multiple sequences, respectively. Note that the apparent length was longer than the nominal length of the FM (5 mm). Table 2 reports the mean, STD and range of the time needed by each observer to perform the FM localisation using single and multiple sequences. The weighted mean over all the observer is 5.8±1.4 min. Note that all the RTTs localised the FMs first using a single and then multiple sequences for all the patients. The RTTs were free to chose the order of patients and whether concluding the procedure first for each the patients using both single and multiple sequences or first for all the patients using single sequence and then repeat for all the patients using multiple modalities. Possible differences in the way the RTTs performed the procedure does not permit to understand whether the FM localisation is faster using single or multiple sequences. In addition, a histogram reporting the frequency of unreliable FM localisation, as perceived by the RTTs is shown in Fig. 1 for four out of five observers; one of the observers did not report the reliability of the localisation. The observers reported the perceived reliability without distinction between localisation performed employing a single and multiple sequences. (PDF 108 kb)

Additional file 3 Single patient investigation. As a supplementary material, we report CT and MRI images for the patients P4 and P6, which were found having LoA >2 mm in maximum one of the three FMs for localisation performed with multiple sequences. Zoom of an axial slice of CT (top left), bSSFP (top centre-left), SPGR (top centre-right) and GRE (top right) images for the patients P4, P6 before image registration as well as schematic representations of the centres of the FMs as localised by all the observers (bottom) are shown in Figs. 1 and 2, respectively. For completeness, we report also the CT ad MRI images along with the schematic representation of the centres of the FM for patient P14 in Fig. 3. Note that this patient was not considered during the analysis since had a hip implant. (PDF 1823 kb)

## Abbreviations

AP: Anterior-posterior
bSSFP: Balanced steady-state free precession
CT: Computed tomography
CM: Centre of mass
FM: Fiducial marker
FOV: Field of view
GRE: Gradient-recalled echo
HU: Hounsfield units
IGRT: Image-guided radiotherapy
IQR: Inter quartile range
kV: Kilo Volt
LoA: Limit of agreement
LoA _CM_: Limit of agreement of the centre of mass
MV: Mega volt
MR(I): Magnetic resonance (imaging)
Obs: Observer
P: Patient
PTV: Planning target volume
RTT: Radiation therapy technician
SPGR: Spoiled gradient-recalled echo
STD: Standard deviation
TE: Echo time
TR: Repetition time
