# Supplementary material for: Evaluation of gold fiducial marker manual localisation for magnetic resonance-only prostate radiotherapy
Source: Radiat Oncol. 2018 Jun 5;13:105. doi: 10.1186/s13014-018-1029-7 (PMC5989467; doi:10.1186/s13014-018-1029-7)
Supplement: Supplementary file 1 — [1.] GeneralGuidelineFMloc.pdf which presents a short description of the procedure; [2.] PracticalInstructionFMloc.pdf which describes step-by-step the procedure; [3.] Checklist_Obs.pdf which is aimed at supporting the RTTs during the procedure in keeping track and annotate for which patient the localisation was found problematic. (ZIP 194 kb) [file 13014_2018_1029_MOESM1_ESM.zip › Additional file 1/Checklist_Obs.pdf]

Which sequence did you use?

Maspero Matteo m.maspero@umcutrecht.nl

Add a x in the column below after saving the data

Patient Nr

**1**	**bSSFP**	**SPGR**	**GRE**
**2**			
**3**			
**4**			
**5**			
**6**			
**7**			
**8**			
**9**			
**10**			
**11**			
**12**			
**13**			
**14**			
**15**			
**16**			
**17**			

Notes

Did the localisation change according to you (Y/N)?*

Please report the Time

*(optional) You can also report if a marker was found diffi

a marker was found difficult to localise
