# Supplementary material for: Evaluation of gold fiducial marker manual localisation for magnetic resonance-only prostate radiotherapy
Source: Radiat Oncol. 2018 Jun 5;13:105. doi: 10.1186/s13014-018-1029-7 (PMC5989467; doi:10.1186/s13014-018-1029-7)
Supplement: Supplementary file 1 — [1.] GeneralGuidelineFMloc.pdf which presents a short description of the procedure; [2.] PracticalInstructionFMloc.pdf which describes step-by-step the procedure; [3.] Checklist_Obs.pdf which is aimed at supporting the RTTs during the procedure in keeping track and annotate for which patient the localisation was found problematic. (ZIP 194 kb) [file 13014_2018_1029_MOESM1_ESM.zip › Additional file 1/GeneralGuidelineFMloc.pdf]

Guidelines for manual detection of gold Fiducial Markers

An inter-observer study

[May 2017 – revised before publication on March 2018, m.maspero@umcutrecht.nl]

Aim

1) Verify the precision of manual FM localization performed by **5** clinically involved RTTs

2) Verify whether the use of multiple sequences increase the agreement/detection rate

Main idea

To detect the Fiducial Markers (FM) along with their orientation using only MRI. The FM localisation should be performed first on the bSSFP sequence (currently used for prostate delineation and also called bTFE SPAIR) and afterwards using also the SPGR (also called T1 3D FFE mDixon) and GRE (mFFE 3D seeds) sequence. If the observer does not feel comfortable he/she can also use other sequences. The sequences available are:

Case

Acquisition date	Modality name	Description	Instance	Matched
2015-09-28 10:19:33.05	mr	t T2 TSE	0	matched
2015-09-28 10:05:39.11	mr	s T2 TSE	0	matched
2015-09-28 10:14:32.27	mr	t T1 3D FFE mDIXON 1...	3	matched
2015-09-28 10:09:21.05	mr	t bTFE SPAIR	0	master
2015-09-28 10:23:58.9	mr	t DWI SPAIR 6bval	0	matched
2015-09-28 10:31:53.09	mr	t mFFE 3D Seeds	0	matched
2015-09-28 10:31:53.09	mr	t mFFE 3D Seeds	1	matched
2015-09-28 10:31:53.09	mr	t mFFE 3D Seeds	2	matched
2015-09-28 10:31:53.09	mr	t mFFE 3D Seeds	3	matched
2015-09-28 10:31:53.09	mr	t mFFE 3D Seeds	4	matched

1) bSSFP or bTFE = used for delineation. This should be your first choice

2) SPGR or T1 3D FFE mDixon = it is used to check for intraprostatic bleedings and it can be useful to understand whether a FM is surrounded by a blood clot.

3) GRE or mFFE 3D Seeds [OPTIONAL sequence] = sequence acquired after ~ 20 min from the bSSFP. It has also complex images, and the FM may have a specific pattern in complex data. (Instance 3 is useful or 1,2)

What is requested to RTT?

FM localization on 17 patients (for a total of 51 Fms) using the bSSFP sequence alone and using an additional sequence. The estimated time per patient is ~ 7.5 min → Max estimated total time is ~ 4 hours

Main step during the manual detection

0) To open the patient and image (first 'bSSFP' only and then using 'bSSFP', 'SPGR' or 'GRE')

1) To window/Level the image intensity such that a standardize image intensity is used and zoom within the FOV to eliminate distraction of the observer during localisation.

2) To click the Top and Bottom of each observed FM. The centre will be afterwards estimated. No order is necessary during the FM detection, and also no preferential definition of top and bottom of a FM.

3) Save the marker location following a name convention E.g. use "FM_1b" to indicate the first FM and the "b" stands for "bottom".

What is available? The study "interobserver gold FM" with the registered images for all the patients
