# Supplementary material for: Evaluation of gold fiducial marker manual localisation for magnetic resonance-only prostate radiotherapy
Source: Radiat Oncol. 2018 Jun 5;13:105. doi: 10.1186/s13014-018-1029-7 (PMC5989467; doi:10.1186/s13014-018-1029-7)
Supplement: Supplementary file 1 — [1.] GeneralGuidelineFMloc.pdf which presents a short description of the procedure; [2.] PracticalInstructionFMloc.pdf which describes step-by-step the procedure; [3.] Checklist_Obs.pdf which is aimed at supporting the RTTs during the procedure in keeping track and annotate for which patient the localisation was found problematic. (ZIP 194 kb) [file 13014_2018_1029_MOESM1_ESM.zip › Additional file 1/PracticalInstructionFMloc.pdf]

Practical Instructions

[May 2017, m.maspero@umcutrecht.nl]

Time estimated per patient: 5 min → Max total time around 4 hours

Preliminary info that can be helpful during the detection

1) The number of marker is 3;

2) The dimension of the FM is fixed: cylindrically shaped with 5mm length and 1 mm diameter. The FMs appear as hypo-intense signal: the dimension of such region is usually larger that the actual size of the FM and it could differ according to the orientation with respect to the FM;

3) The FMs are inserted with only 2 needle insertions. This means that for one needle 2 FMs are released along a line, or, in other words, 2 FMs should have a transverse consistent position (within 5 mm).

N.B. it may occur that 2 of the FMs released during one needle insertion are positioned close to each others and are not distinguishable.

Solution: The user may keep track of the size (you can count the number of slices) in the Feet/Head direction to verify if the size of the signal void is too large to contain only one FM.

4) If FM detection is performed using a different sequence, **intra-fraction** migration could occur and this means that the location of the FM may be inconsistent. The ground truth should be the position of the FM on the sequence used for prostate delineation (bSSFP), then use first of all the GRE sequence in combination with bSSFP.

Preliminary Check/Test Patient

0.a) Decide where you wish to save the data (create a dedicated folder in the pc?), and save all the marker locations in the same folder (specifying the ptnumber); You should also keep the data saved in the study, but it is good to have also a local version.

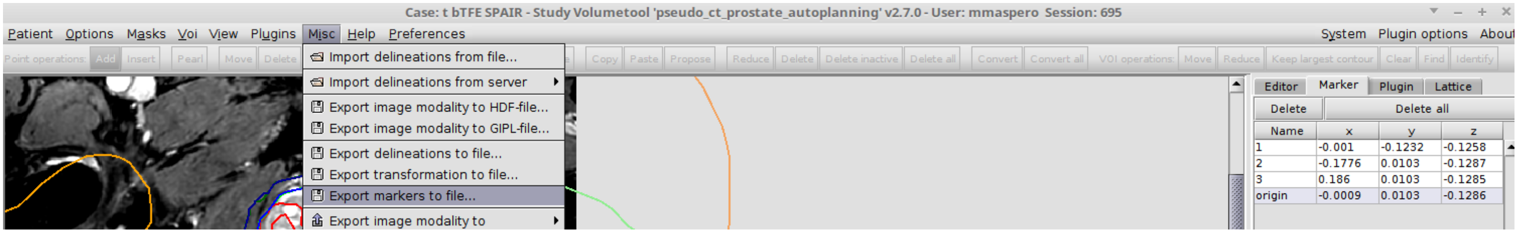

You can locally save the location of the marker from “Misc/Export Marker to file...”. As show in the figure above the second tab on the right shown the locations of the marker.

2) Once the localisation is performed zip the folder and send it to the dedicated reseracher.

The actual procedure for each patient

0) in “studyvolumetool” select the study “interobserver gold FM” and

a) **Open the “bSSFP” sequence of a patient** (e.g. Pt001)**.** Select the study “Obs#_intials” where # will be an assigned number and initials are the initials of your name [if you cannot find ask Matteo and he will make it ;)].

b) Standardize the **window/levelling**. The proposal is to set the windows for the bSSFP to max 1200 and min 0, while level to 800. First set min/max and then the level. N.B. If you do not feel comfortable it is possible to adjust differently the W/L!

c) **Zoom** keeping the prostate in the center of the visualisation. A good value for the zoom may between 2/4.

d) **Scout localization**. Scroll in the transverse plane to have a first idea where the potential FM could be. Check also in the coronal and sagittal plane the position of the FM.

e) **Detect the Top and Bottom** of each visible FM. Please start from the FM that you feel more confident and try to detect always 3 FM:

i. Scroll through the transverse plane and position the pointer in the middle of the FM;

ii. Identify the top and bottom of each FM using the transverse, but also the coronal plane; Position the cursor on the location and save the position by naming it in the dedicated tab (see presentation).

iii. <u>Name convention</u>. Name the marker in the dedicated plugin (right column, 2nd tab) using 'FM' followed by an incremental number from 1 to 3 (or 4) and followed by '_b' or '_t' (please do not forget the underscore '_') to indicate whether is the top or bottom of the marker (order of top and bottom are not important!). E.g. for the first marker you will use the name '1_t' for the top, and '1_b' for the bottom;

f) S**ave the file and the patient** with the position of all the FMs from the bTFE sequence. In the tab 'Misc', you can find the export marker position. Call the file “Pt###.gof”, where ### is the incremental number correspondent to the patient selected or PatientID. E.g. for patient 1 → Pt001.gof

g) Open the study Obs#_initialsMORE and select the **GRE sequence** window/level and adjust the position of the FMs if needed. W/L might be for the FFE [0;1500] and zoom between 2 and 4.

Note that on GRE bright area in the prostate should correspond to bleeding. It may happen that a FM is in one of these region. Please report in the **excel file** (or print it ;) and write) whether you think that the use of the GRE changed your FM localisation. Be aware of the fact that **intra-fraction motion** could occur and that the absolute position of the FM could be slightly different among sequences.

h) Locate all the FMs using the bSSFP as a reference sequence and the GRE as help, if you still do not feel comfortable during the localisation please annotate. In this case you are allowed also the detect more or less FM.

i) **save the file** containing all the positions of all FMs from the GRE sequence. In the tab 'Misc', you can find the export marker position. Call the file “Pt###_T1.gof”, where ### is the incremental number correspondent to the patient selected or PatientID. E.g. for patient 1 → Pt001_T1.gof

j) Save the patient, and open the next one and repeat from point a) to j)
