# Supplementary material for: Evaluation of gold fiducial marker manual localisation for magnetic resonance-only prostate radiotherapy
Source: Radiat Oncol. 2018 Jun 5;13:105. doi: 10.1186/s13014-018-1029-7 (PMC5989467; doi:10.1186/s13014-018-1029-7)

RESEARCH

Evaluation of gold Fiducial Marker manual localisation for Magnetic Resonance-only prostate Radiotherapy

Matteo Maspero*, Peter R Seevinck (p.seevinck@umcutrecht.nl), Nicole J Willems (n.willems-2@umcutrecht.nl), Gonda G Sikkes (g.g.sikkes@umcutrecht.nl), Geja J de Kogel (G.deKogel@umcutrecht.nl), Hans CJ de Boer (J.C.J.deBoer-6@umcutrecht.nl), Jochem RN van der Voort van Zyp (J.R.N.vanderVoortvanZyp@umcutrecht.nl) and Cornelis AT van den Berg (c.a.t.vandenBerg@umcutrecht.nl)

*Correspondence: m.maspero@umcutrecht.nl, matteo.maspero.it@gmail.com
Universitair Medisch Centrum Utrecht, Heidelberglaan 100, 3508 GA Utrecht, The Netherlands
Full list of author information is available at the end of the article

RESEARCH

Additional file 2 — Annotations on the FM localisation

As part of the supplementary material, we report the apparent length of the FMs for each observer and the time spent by each observer performing the FM localisation over all the patients. In particular, table 1 shows the mean, standard deviation (STD), range [min, max] of the apparent length, expressed in mm. The weighted mean over all the observer is 7.5 ± 0.6 mm and 7.7 ± 0.7 mm for localisation using a single and multiple sequences, respectively. Note that the apparent length was longer than the nominal length of the FM (5 mm). Table 2 reports the mean, STD and range of the time needed by each observer to perform the FM localisation using single and multiple sequences. The weighted mean over all the observer is 5.8 ± 1.4 min. Note that all the RTTs localised the FMs first using a single and then multiple sequences for all the patients. The RTTs were free to chose the order of patients and whether concluding the procedure first for each the patients using both single and multiple sequences or first for all the patients using single sequence and then repeat for all the patients using multiple modalities. Possible differences in the way the RTTs performed the procedure does not permit to understand whether the FM localisation is faster using single or multiple sequences. In addition, a histogram reporting the frequency of unreliable FM localisation, as perceived by the RTTs is shown in figure 1 for four out of five observers; one of the observers did not report the reliability of the localisation. The observers reported the perceived reliability without distinction between localisation performed employing a single and multiple sequences.

Table 1 **Apparent detection length of the FMs as obtained performing the localisation using a single (bSSFP) of multiple (bSSFP, SPGR and GRE) sequences for each observer. All the values are expressed in mm.**

Sequence	Observer	mean	STD	Range
	1	8.3	1.9	[3.1, 15.2]
	2	7.6	1.4	[5.0, 13.2]
**Single**	3	7.1	1.3	[3.0, 9.2]
	4	7.4	1.8	[3.2, 13.3]
	5	7.8	1.6	[4.1, 15.2]
	1	8.3	1.6	[4.1, 14.2]
	2	7.7	1.5	[4.0, 14.2]
**Multiple**	3	7.2	1.3	[3.0, 9.1]
	4	7.4	1.8	[3.2, 13.3]
	5	7.7	1.2	[4.0, 10.0]

Table 2 **The mean, standard deviation (STD) and maximum time needed for the FM localisation procedure of each observer over all the FMs using single or multiple sequences, expressed in minutes.**

Observer	Single			Multiple		
	mean	STD	Maximum	mean	STD	Maximum
**1**	8.9	3.3	15	8.5	3.6	15
**2**	8.8	4.1	15	7.1	2.4	11
**3**	4.3	1.9	8	4.5	3.1	10
**4**	6.6	4.7	16	4.8	3.7	14
**5**	6.2	3.9	13	5.3	3.4	13

Figure 1 Frequency of reported unreliable FM localisation according to four out of five observers. Note that patient P14 had hip implant and the results are here presented but were excluded in the statistical analysis.

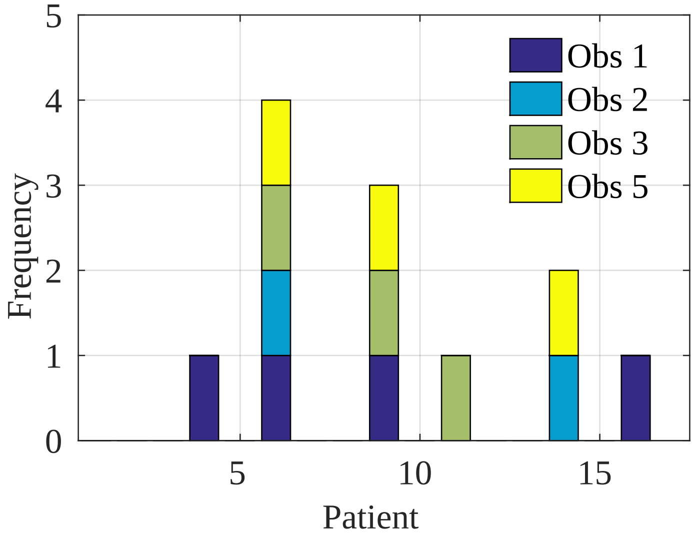

Supplement: Supplementary file 2 — Annotations on the FM localisation. As part of the supplementary material, we report the apparent length of the FMs for each observer and the time spent by each observer performing the FM localisation over all the patients. In particular, Table 1 shows the mean, standard deviation (STD), range [min, max] of the apparent length, expressed in mm. The weighted mean over all the observer is 7.5±0.6 mm and 7.7±0.7 mm for localisation using a single and multiple sequences, respectively. Note that the apparent length was longer than the nominal length of the FM (5 mm). Table 2 reports the mean, STD and range of the time needed by each observer to perform the FM localisation using single and multiple sequences. The weighted mean over all the observer is 5.8±1.4 min. Note that all the RTTs localised the FMs first using a single and then multiple sequences for all the patients. The RTTs were free to chose the order of patients and whether concluding the procedure first for each the patients using both single and multiple sequences or first for all the patients using single sequence and then repeat for all the patients using multiple modalities. Possible differences in the way the RTTs performed the procedure does not permit to understand whether the FM localisation is faster using single or multiple sequences. In addition, a histogram reporting the frequency of unreliable FM localisation, as perceived by the RTTs is shown in Fig. 1 for four out of five observers; one of the observers did not report the reliability of the localisation. The observers reported the perceived reliability without distinction between localisation performed employing a single and multiple sequences. (PDF 108 kb) [file 13014_2018_1029_MOESM2_ESM.pdf]
