# Supplementary material for: Evaluation of gold fiducial marker manual localisation for magnetic resonance-only prostate radiotherapy
Source: Radiat Oncol. 2018 Jun 5;13:105. doi: 10.1186/s13014-018-1029-7 (PMC5989467; doi:10.1186/s13014-018-1029-7)
Supplement: Supplementary file 3 — Single patient investigation. As a supplementary material, we report CT and MRI images for the patients P4 and P6, which were found having LoA >2 mm in maximum one of the three FMs for localisation performed with multiple sequences. Zoom of an axial slice of CT (top left), bSSFP (top centre-left), SPGR (top centre-right) and GRE (top right) images for the patients P4, P6 before image registration as well as schematic representations of the centres of the FMs as localised by all the observers (bottom) are shown in Figs. 1 and 2, respectively. For completeness, we report also the CT ad MRI images along with the schematic representation of the centres of the FM for patient P14 in Fig. 3. Note that this patient was not considered during the analysis since had a hip implant. (PDF 1823 kb) [file 13014_2018_1029_MOESM3_ESM.pdf]

RESEARCH

Evaluation of gold Fiducial Marker manual localisation for Magnetic Resonance-only prostate Radiotherapy

Matteo Maspero*, Peter R Seevinck (p.seevinck@umcutrecht.nl), Nicole J Willems (n.willems-2@umcutrecht.nl), Gonda G Sikkes (g.g.sikkes@umcutrecht.nl), Geja J de Kogel (G.deKogel@umcutrecht.nl), Hans CJ de Boer (J.C.J.deBoer-6@umcutrecht.nl), Jochem RN van der Voort van Zyp (J.R.N.vanderVoortvanZyp@umcutrecht.nl) and Cornelis AT van den Berg (c.a.t.vandenBerg@umcutrecht.nl)

*Correspondence: m.maspero@umcutrecht.nl, matteo.maspero.it@gmail.com
Universitair Medisch Centrum Utrecht, Heidelberglaan 100, 3508 GA Utrecht, The Netherlands
Full list of author information is available at the end of the article

RESEARCH

Additional file 3 — Single patient investigation

As a supplementary material, we report CT and MRI images for the patients P4 and P6, which were found having LoA > 2 mm in maximum one of the three FMs for localisation performed with multiple sequences. Zoom of an axial slice of CT (top left), bSSFP (top centre-left), SPGR (top centre-right) and GRE (top right) images for the patients P4, P6 before image registration as well as schematic representations of the centres of the FMs as localised by all the observers (bottom) are shown in figure 1 and figure 2, respectively. For completeness, we report also the CT ad MRI images along with the schematic representation of the centres of the FM for patient P14 in figure 3. Note that this patient was not considered during the analysis since had a hip implant.

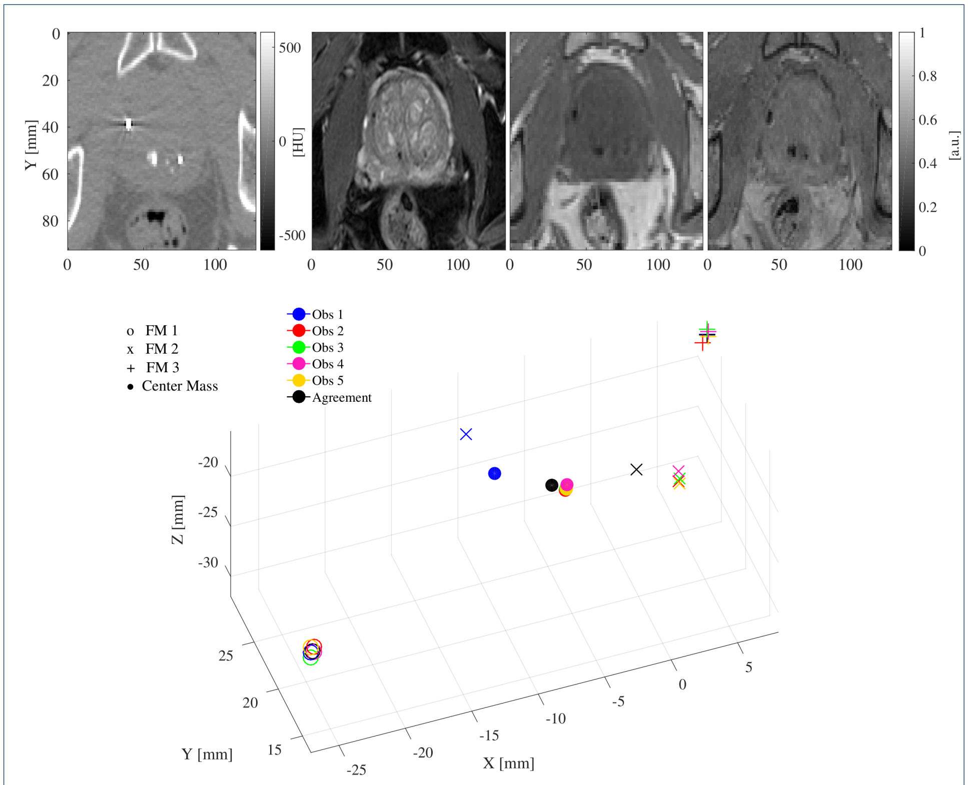

Figure 1 (Top) Zoom of an axial slice of CT (left), bSSFP (centre-left), SPGR (centre-right) and GRE (right) images for patient P4 before image registration. The axes X and Y indicate the anterior-posterior and right-left directions. The intensity of CT image is in Hounsfield Units (HU), while of MR images is normalised to the maximum over the whole dataset. (Bottom) Schematic representation of the centres of the FMs as localised by all the observers for patient P4 using multiple sequences (bSSFP, SPGR and GRE). The labelling of the FM is indicated by the marker: ○, x, + and • for FM having number 1, 2, 3 and for agreement position, respectively. For this patient (P4), the $\mathrm{LoA}_{\mathrm{CM}}$ for FM2 was 8.73, 0.87 and 6.17 mm in X, Y and Z, respectively, which was considered clinically unacceptable.

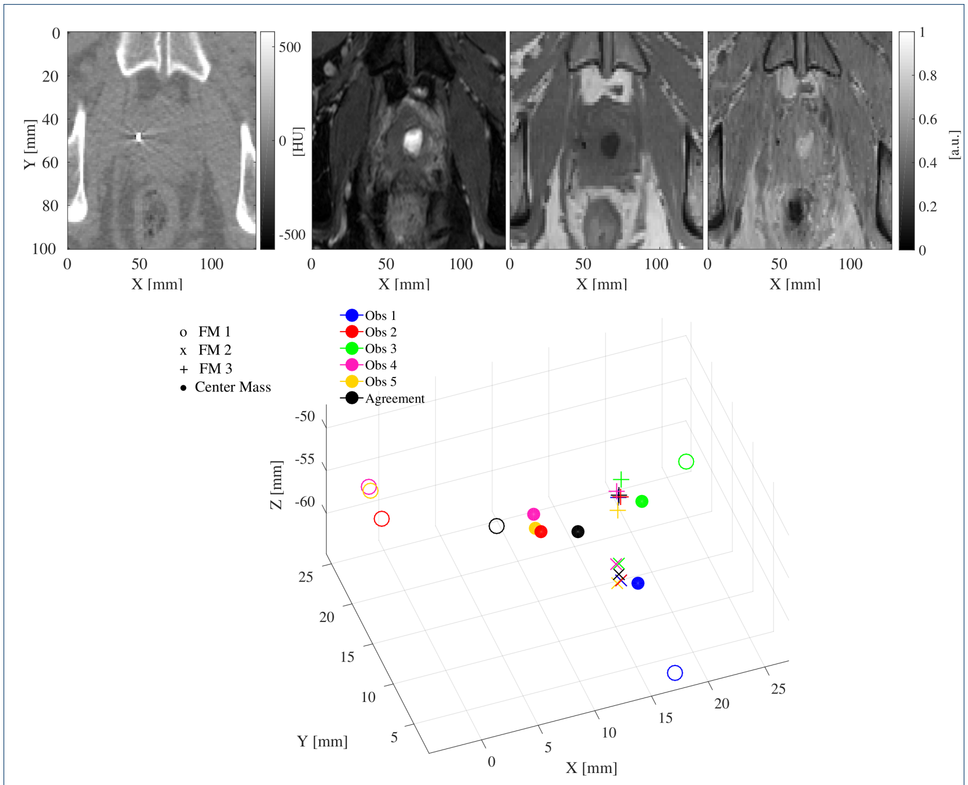

Figure 2 (Top) Zoom of an axial slice of CT (left), bSSFP (centre-left), SPGR (centre-right) and GRE (right) images for patient P6 before image registration. The axes X and Y indicate the anterior-posterior and right-left directions. The intensity of CT image is in Hounsfield Units (HU), while of MR images is normalised to the maximum over the whole dataset. (Bottom) Schematic representation of the centres of the FMs as localised by all the observers for patient P6 using multiple sequences. The labelling of the FM is indicated by the marker: ○, x, + and • for FM having number 1, 2, 3 and for agreement position, respectively. For this patient (P6), the LoA_{CM} for FM1 was 8.99, 12.19 and 3.89 mm in X, Y and Z, respectively, which was considered clinically unacceptable.

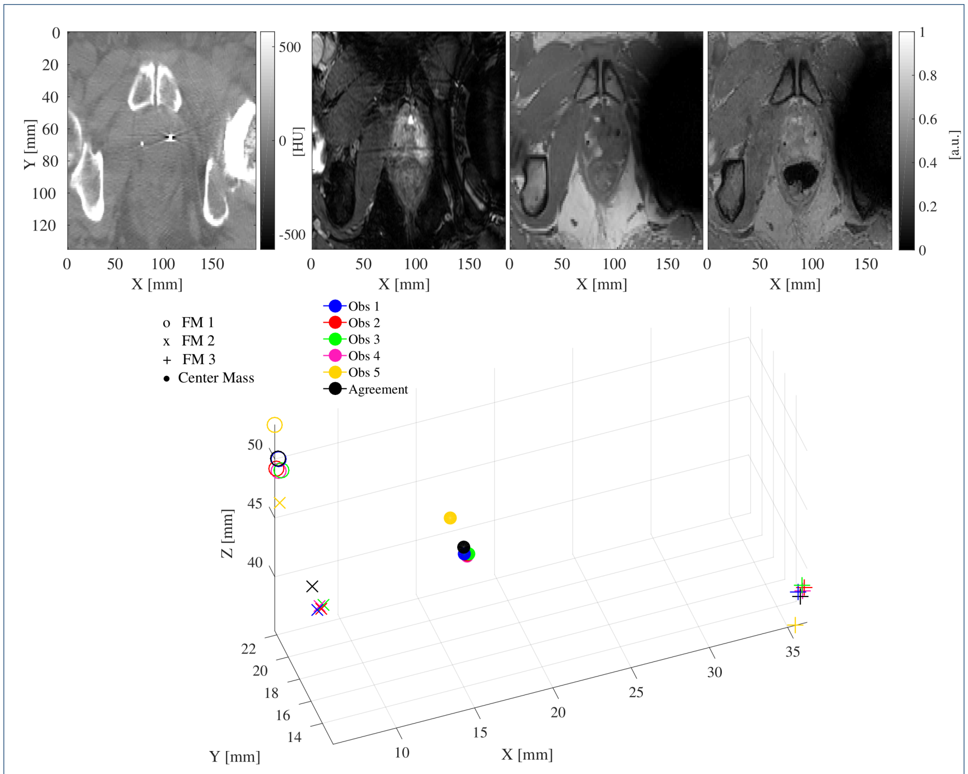

Figure 3 (Top) Zoom of an axial slice of CT (left), bSSFP (centre-left), SPGR (centre-right) and GRE (right) images for patient P14 before image registration. The axes X and Y indicate the anterior-posterior and right-left directions. The intensity of CT image is in Hounsfield Units (HU), while of MR images is normalised to the maximum over the whole dataset. (Bottom) Schematic representation of the centres of the FMs as localised by all the observers for patient P14 using multiple sequences. The labelling of the FM is indicated by the marker: ○, x, + and • for FM having number 1, 2, 3 and for agreement position, respectively. For this patient (P14), the $\mathrm{LoA_{CM}}$ for FM2 was 0.84, 1.62 and 3.42 mm in X, Y and Z, respectively, which was considered clinically unacceptable. The statistics relative to this patients have not been considered in the study due to the presence of the hip implant.
